# Multisensory Integration and Attention in Autism Spectrum Disorder: Evidence from Event-Related Potentials

**DOI:** 10.1371/journal.pone.0024196

**Published:** 2011-08-24

**Authors:** Maurice J. C. M. Magnée, Beatrice de Gelder, Herman van Engeland, Chantal Kemner

**Affiliations:** 1 Department of Child and Adolescent Psychiatry, Rudolf Magnus Institute of Neuroscience, University Medical Center Utrecht, Utrecht, The Netherlands; 2 Laboratory of Cognitive and Affective Neuroscience, Tilburg University, Tilburg, The Netherlands; 3 Martinos Center for Biomedical Imaging, Massachusetts General Hospital and Harvard Medical School, Charlestown, Massachusetts, United States of America; 4 Department of Developmental Psychology, Utrecht University, Utrecht, The Netherlands; Federal University of Rio de Janeiro, Brazil

## Abstract

Successful integration of various simultaneously perceived perceptual signals is crucial for social behavior. Recent findings indicate that this multisensory integration (MSI) can be modulated by attention. Theories of Autism Spectrum Disorders (ASDs) suggest that MSI is affected in this population while it remains unclear to what extent this is related to impairments in attentional capacity. In the present study Event-related potentials (ERPs) following emotionally congruent and incongruent face-voice pairs were measured in 23 high-functioning, adult ASD individuals and 24 age- and IQ-matched controls. MSI was studied while the attention of the participants was manipulated. ERPs were measured at typical auditory and visual processing peaks, namely, P2 and N170. While controls showed MSI during divided attention and easy selective attention tasks, individuals with ASD showed MSI during easy selective attention tasks only. It was concluded that individuals with ASD are able to process multisensory emotional stimuli, but this is differently modulated by attention mechanisms in these participants, especially those associated with divided attention. This atypical interaction between attention and MSI is also relevant to treatment strategies, with training of multisensory attentional control possibly being more beneficial than conventional sensory integration therapy.

## Introduction

In a social environment, events typically involve stimulation through multiple sensory modalities. Multisensory integration (MSI) of these stimuli enables better understanding of the social intentions of others [1], which is of particular importance for perception of visual and auditory emotional stimuli [2]. Several studies yielded suggestions that individuals with Autism Spectrum Disorders (ASD) have difficulty with integrating information across auditory and visual modalities, which suggests that MSI impairments may have an important role in the atypical social behavior of individuals with ASD [3], [4], [5]. Recent work, however, clearly showed that differences in MSI between ASD and typically developing individuals are secondary to the presence of environmental noise, suggesting a differential influence of noise on MSI in this population [6].

Another potential contribution to presumed differences in MSI might come from recent findings indicating that in typically developing individuals MSI can be modulated by attention [7]. MSI is known to occur at multiple stages of processing and is thought to interact with attention depending on what level of processing the integration takes place [8]. At low, pre-attentive levels MSI can automatically capture attention, which is for instance shown by means of faster detection of visual objects through auditory signals [9]. At higher levels top-down attention can facilitate MSI and as such lead to a further spread of attention across modalities [10].

This interaction between MSI and attention is of particular importance, given that individuals with ASD show attentional impairments, particularly when they need to shift attention between auditory and visual modalities [11]. Thus the atypical behavior and brain activation seen in individuals with ASD during the performance of tasks designed to study MSI could in fact reflect problems with attentional focus. To understand the neurocognitive mechanisms underlying atypical social interaction in ASD, it is important to determine the role of attention in MSI in this group.

In the present study, we looked at the MSI of emotional (happy and fearful) faces and voices. We measured event-related potentials (ERPs) and focused on two peaks in the ERP signal that are sensitive to MSI, namely the auditory P2 and the visual N170. The frontal–central P2 peak, which is known to reflect activity from auditory cortical areas, is sensitive to the congruency between emotions conveyed by facial expression and the voice [12]. The N170 is a negative deflection around 170 ms at bilateral occipital-temporal sites and is associated with the structural encoding of faces [13]. Previous research has shown that this predominantly visual processing area is also sensitive to the congruency of cross-modal emotions [4], [14]. We examined MSI by presenting both modality-specific and cross-modal stimuli and analyzed data in two ways. First, we compared ERPs in response to audiovisual (AV) stimuli with the sum of ERPs in response to unisensory stimuli (face only + voice only). Differences in the ERP scores for these two situations (AV - (A+V)) are attributed to the interaction between the two modalities and are thought to reflect lower-order MSI because ERPs are not affected by the content of the stimulus [15]. Second, we explored higher-order MSI by contrasting emotionally congruent and incongruent AV conditions. Differences in ERPs in response to these stimuli provide clear evidence of higher-order MSI, as a mismatch can only be detected after recognition of the unisensory input and its functional integration [12], [16].

We investigated how manipulation of attention affected the integration of visual and auditory emotional information. To this end, participants were presented with emotional faces and voices while using distracters to manipulate attention to the faces and voices. We hypothesized that atypical MSI in individuals with ASD would be secondary to manipulations of attention.

## Materials and Methods

### Ethics Statement

Written informed consent was obtained from each participant before the session, according to the Declaration of Helsinki (2008). The Medical Ethics Committee of the University Medical Center Utrecht approved the study.

### Participants

Twenty-three high-functioning adult males with ASD (five left-handed) and 24 typically developing adult male controls (seven left-handed) participated in the study. All individuals were administered the Wechsler Adult Intelligence Scale, Dutch edition (WAIS-III-NL). Mean age and total IQ scores were statistically similar for individuals with ASD (average age 22.7 years, SD 3.8; IQ 118.2, SD 10.8) and individuals from the control group (average age 22.7, SD 1.9.; IQ 116.1, SD 10.6). All individuals with ASD reached diagnostic thresholds on all domains of the ADOS [17] and ADI-R [18]. All participants were free of seizure disorders, neurological diseases, or head trauma. Additionally, before assigning individuals to the control group they were screened negative for psychiatric complaints, substance abuse and familial history of psychiatric disorders. They were all paid for their participation. Written informed consent was obtained from each participant before the session, according to the Declaration of Helsinki (2008). The Medical Ethics Committee of the University Medical Center Utrecht approved the study.

### Stimuli and Procedure

Visual stimuli consisted of 12 happy and 12 fearful faces (6 male and 6 female faces) taken from the Karolinska Directed Emotional Faces set [19]. Auditory stimuli consisted of 12 happy (laughing) and 12 fearful (gasping) vocalizations. Each visual stimulus was combined with an auditory fragment in order to construct AV stimulus pairs with either a congruent or an incongruent affective content. The pictures of faces were 19 cm height by 13 cm width, which were presented at a viewing distance of 80 cm. The auditory stimuli were presented binaurally through stereo insert earphones (Eartone ABR) at a level of 83 dB(a). Mean levels for sound and luminance were equal across stimuli.

**Figure 1:**
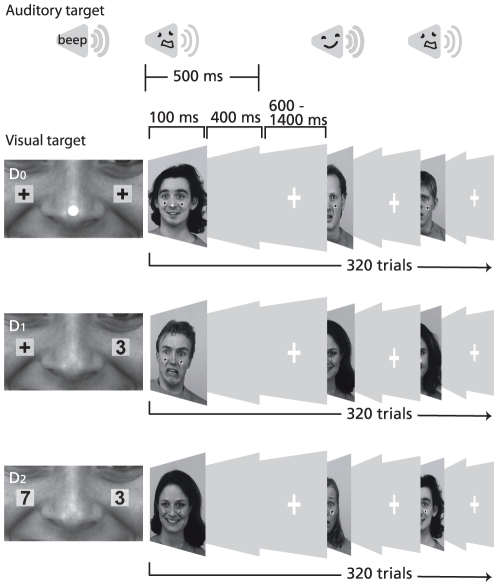
Layout of the task. Attention was manipulated between AV blocks, and participants had to respond to a visual dot and auditory beep (D_0_), a single digit ‘3’ (D_1_), or when two digits together add up to 10 (D_2_). Stimuli were presented concurrently during AV blocks and in isolation during unisensory blocks.

Audiovisual, auditory, and visual trials were randomly presented in three separate blocks. Both unisensory blocks consisted of 160 repetitions of happy and fearful stimuli. During AV blocks, visual and auditory stimuli were presented concurrently and consisted of four stimulus categories: congruent audiovisual happy, congruent audiovisual fear, incongruent visual fear-auditory happy and incongruent visual happy-auditory fear. Each AV stimulus combination was presented 80 times, resulting in a total of 320 stimulus repetitions. Attention was manipulated between blocks, containing divided attention, easy-, and hard selective attention conditions (Figure 1).

Visual stimulus duration was 100 ms, followed by a gray screen for 400 ms. Auditory stimulus duration was 500 ms. The shorter visual stimulus duration was chosen to optimize the manipulation of attention. The intertrial interval varied randomly between 600 – 1400 ms, during which a central fixation cross was presented on screen.

Target trials were introduced in order to ensure that participants paid attention to the stimuli. In visual target trials, a small white dot was positioned on the nose of the face for 85 ms. In auditory target trials, a 1000-Hz tone (83 dB(a); fade-in and fade-out of 10 ms respectively) was presented together with the voice stimulus for 50 ms. In AV divided attention blocks, both visual and auditory targets occurred one by one in random order, and participants had to attend to both. Each block contained 10% of target trials, except the AV easy- and hard-selective attention conditions. Participants were instructed to push a designated button every time a target trial occurred. All target trials were excluded from further analyses.

### Attention manipulation

In the unisensory conditions participants were required to attend to either visual or auditory targets. For the AV stimuli, three attentional conditions were included. In the divided attention condition (D_0_), participants were instructed to attend to V and A stimuli, and respond to both visual and auditory targets, which were presented in 10% of all stimuli. In the two selective attention conditions, attention was directed by placing task-relevant stimuli on the cheeks of the face picture. In the easy (D_1_) condition, participants were instructed to ignore the faces and voices, and to attend only to a single digit (0 through 9) that was randomly shown on either the left or right cheek of the face picture. They were instructed to respond only to digit ‘3’, which was presented in 10% of all stimuli. In the hard (D_2_) condition, participants were instructed to attend only to the serial presentation of two digits (0 through 9), with one presented on either cheek of the face picture. They had to respond only when the two digits presented together added up to 10, which was the case in 10% of all stimuli.

### Recordings

EEGs were recorded at a sample rate of 2048 Hz from 64 locations using standard Ag/AgCl pin-type active electrodes (BIOSEMI, Amsterdam, the Netherlands) mounted in an elastic cap, referenced to an additional active electrode (Common Mode Sense) during recording. EEG signals were band-pass filtered (1–30 Hz, and an additional 50 Hz notch filter) off-line and re-referenced to an average reference. Horizontal and vertical EOGs were measured for offline correction. The raw data were segmented into epochs for visual, auditory, and AV categories separately, using Brain Vision Analyzer (Brain Products GmbH, Gilching, Germany). All categories consisted of 1000-ms epochs, including a 100-ms pre-stimulus baseline. After EOG correction, epochs with amplitudes exceeding ±100 µV at any channel were automatically rejected. Lowest allowed activity was 3 µV/ 200 ms, and the maximal allowed voltage step per sampling point was 50 µV.

### Data analyses

The effects of the various manipulations on the auditory P2 and the visual N170 signals were measured. Because of the known multisensory effects of these peaks and because of clarity in the present article, we chose not to look for possible other MSI effects. For reasons of readability, we further decided to report significant results only.

The auditory P2 was measured at frontal-central electrodes (FC1, FC2, FCz) as the mean of the individual peak amplitudes over the three electrodes between 150 and 230 ms. The visual N170 was measured at bilateral occipital-temporal electrodes (P7, P8), between 130 and 210 ms. These electrodes and time intervals were selected based on visual inspection of the grand averaged waveforms, after which an automated procedure was used to identify individual peaks. First, we tested the effect of emotion on auditory and visual stimuli separately, to be able to differentiate any group effects in this respect from possible MSI effects. This unisensory analysis consisted of the between-subjects factor Group (ASD vs. control group), and the within-subjects factor Emotion (happy vs. fear). Additionally, the N170 analysis included an extra within-subjects factor Hemisphere (left vs. right).

Second, we tested lower-order MSI effects by comparing ERPs in response to AV stimuli to the sum of ERP signals obtained in unisensory conditions (A+V). If AV responses do not equal the sum of unisensory auditory and visual evoked potentials, this is considered a neural correlate of MSI [20]. Possible confounder processes like anticipatory slow wave potentials are cancelled out by using variable intertrial intervals and high-pass filters of 1 Hz [21]. The additive model might possibly lead to spurious interaction effects on early (20–40 ms) and late ERPs (P3 peak). Analyzing mid-range ERP components only (N170 and P2) further reduces the impact of these potential confounders. Our use of a detection task and discarding the target stimuli is further known to reduce other confounding factors such as motor-response-related ERP activity, as described in [22]. Our analyses consisted of the between-subjects factor Group (ASD vs. control group) and the within-subjects factors Presentation (AV vs. A+V), and Emotion (happy vs. fearful). N170 analyses included an extra within-subjects factor Hemisphere (left vs. right).

Third, we compared AV congruent and incongruent conditions in order to measure higher-order MSI. Differences in ERPs in response to these stimuli provide clear evidence of higher-order MSI, as a mismatch can only be detected after recognition of the unisensory input and its functional integration [12], [16]. For this comparison, analyses consisted of the within-subjects factors Emotion (happy vs. fearful), Congruency (congruent vs. incongruent), and Attention (D_0_, D_1_, D_2_). N170 effects included an additional within-subjects factor Hemisphere (left vs. right).

## Results

### Behavioral data

Independent-samples *t*-tests on target trials in unisensory conditions showed that visual and auditory target trials were detected almost faultlessly in both groups. However, individuals with ASD made significantly more errors (false-positives and misses) with AV stimuli than controls (D_0_ average of 4.0 vs. 0.92 errors, *t*(45) = −2.7, *p*<0.01; D_1_ 1.65 vs. 0.46 errors, *t*(45) = −2.3, *p*<0.05; D_2_ 3.2 vs. 1.5 errors *t*(45) = −2.1, *p*<0.05).

### Electrophysiological data for unisensory conditions

No effects of attention were found, and no significant differences between groups were found regarding the effects of happy and fearful emotions on unisensory conditions.

### Electrophysiological data for lower-order integration

**Figure 2:**
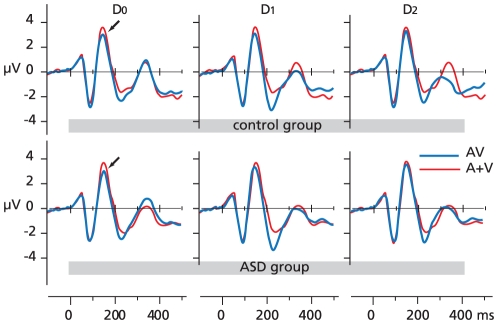
Frontal-central P2 amplitudes. Frontal-central P2 peaks are significantly larger (p<.001) to the sum of ERPs to unisensory stimuli (A+V) than to cross-modal (AV) stimuli under divided attention (D_0_) and easy selective attention conditions (D_1_) for both groups.

P2 amplitudes were larger with the sum of ERPs to unisensory stimuli (A+V) than with multisensory (AV) stimuli under divided attention (D_0_; *F*(1,45) = 17, *p*<0.001) and easy selective attention (D_1_; *F*(1,45) = 5.2, *p*<0.001) conditions. No P2 latency effects were found for lower-order integration analyses (Figure 2). N170 amplitudes were larger in response to A+V stimuli than in response to AV stimuli under the divided attention condition only (*F*(1,45) = 15, *p*<0.001). No significant differences between groups were found and no significant lower-order integration effects were found for N170 latencies.

### Electrophysiological data for higher-order integration

Incongruent fearful visual stimuli resulted in a significant increase in auditory P2 amplitudes compared to congruent stimuli (*F*(1,46) = 5.7, *p*<0.05). In addition, congruent happy visual stimuli resulted in significantly larger amplitudes of the auditory P2 compared to incongruent stimuli (*F*(1,46) = 5.2, *p*<0.05). Further, a main effect of attention was noted, as P2 amplitudes were significantly smaller in divided attention conditions than easy and hard selective attention conditions (*F*(1,46) = 3.4, *p*<0.05). Congruency analyses did not show any effects on P2 latencies.

**Figure 3:**
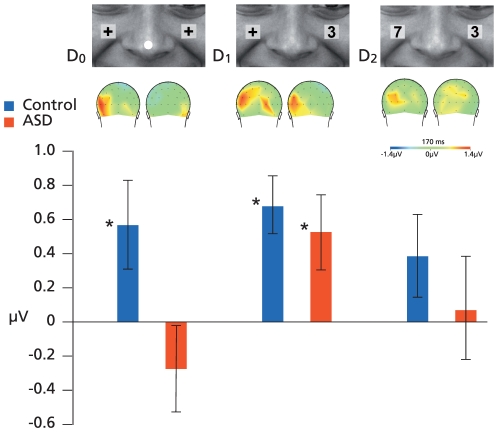
Occipital-temporal N170 amplitudes. Left N170 amplitude differences (±SE) between stimulus conditions (FF - FH) show a lack of higher-order MSI in the D_0_ condition for ASD individuals, while both groups show such an effect in the D_1_ condition (* = p<.05).

**Figure 4:**
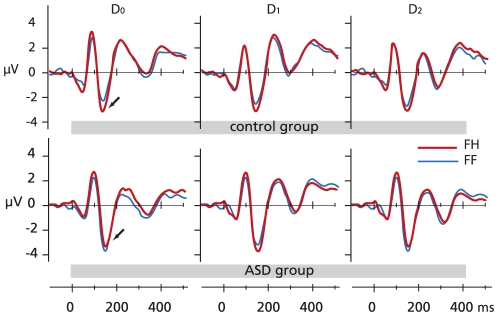
Event Related potentials. ERPs at P7 electrode to congruent visual – auditory fear (FF) and incongruent visual fear – auditory happy (FH) stimuli in the D_0_, D_1_ and D_2_ conditions from the control group (above) and the ASD group (below). The arrows point to the N170 amplitudes.

The amplitude of N170 was larger when fearful visual stimuli were accompanied by incongruent rather than congruent auditory input (*F*(1,46) = 5.9, *p*<0.05). This effect differed between Groups (*F*(1,46) = 5.1, *p*<0.05), as significant congruency effects were seen for divided attention conditions in the left hemisphere in the control group (*t*(23) = 2.2, *p*<0.05), but not in the ASD group (*t*(22) = −1, *p* = NS; Figures 3 & 4). With easy selective attention conditions we found significant congruency effects for fearful stimuli in both groups (*t*(23) = 4.1, *p*<0.05 in the control group; *t*(22) = 2.4, *p*<0.05 in the ASD group) but no congruency effects with hard selective attention conditions in either group. Congruency analyses did not show any effects on N170 latencies.

## Discussion

Our goal was to study the effect of attention on the MSI of emotional signals in typically developing individuals and individuals with ASD in order to determine whether the poor performance of individuals with ASD on tasks involving MSI is indeed the result of a deficit in MSI or the result of abnormalities in directed attention. We measured lower-order and higher-order MSI, using ERPs. Because unisensory processing in ASD might be atypical and thus influence MSI, we tested for group differences in ERP amplitudes during unisensory auditory and visual processing but found no such differences. Lower-order MSI was defined by smaller auditory P2 and visual N170 amplitudes in response to multisensory (AV) conditions as compared to the combined ERP response to unisensory (A+V) stimuli. Lower-order MSI was most apparent when attention was divided between auditory and visual components of the stimulus. Importantly, and in accordance with earlier studies [5], [23], [24], this lower-order MSI was shown to be intact in individuals with ASD.

Second, we explored higher-order MSI by contrasting emotionally congruent and incongruent face-voice pairs. ERP activity related to both visual and auditory processing was affected by cross-sensory incongruence. This congruency effect was observed clearly in the control group but not in the ASD group when attention was divided between the visual and auditory components of the stimulus. However, both selective attention conditions triggered similar AV congruency effects in both groups, namely, an effect in the easy, but not in the hard, condition.

This indicates that individuals with ASD are indeed able to integrate facial and auditory information at a high level of cognitive processing. These data are in line with the results of a recent study [25], in which two-year old children with ASD were found to be highly sensitive to the synchrony between point-light displays of biological motion and speech sounds, indicating that MSI was intact. However, the lack of MSI among individuals with ASD in the divided attention condition in the present study indicates that attention is an important factor in the integration of faces and voices in this group. Attentional impairments are among the most consistently reported cognitive deficits in ASD and are considered a core deficit of the disorder [11]. Several studies have indicated that individuals with ASD have problems with tasks that involve paying attention to different modalities [26], [27]. On the basis of our data, we suggest that while there is no reason to assume primary deficits in MSI in ASD, many studies will report such impairments because the ability to divide attention over information from different modalities is abnormal in this group.

Attention is known to have a differential effect on MSI depending on the level of processing at which the integration takes place [8]. Bottom-up mechanisms, induced by MSI, can for instance capture attention while on the other hand top-down attention can facilitate the integration of cross-modal inputs [10]. Our findings correspond with the notion of multisensory congruency matching being a relatively higher form of MSI that is more sensitive to attention. As such, this interaction between higher-order MSI and attention might be more sensitive to failure in ASD, and possibly also in other clinical syndromes such as schizophrenia [28].

The absence of convincing evidence for multisensory dysfunction directly questions the usefulness of sensory integration therapies in individuals with ASD. In line with earlier reports disputing on the effectiveness of sensory integration therapies in ASD [29], we recommend that more research is imperative to determine the most effective types of interventions in this area. Based on our data, treatment strategies may focus on the training of multisensory *attentional* control rather than conventional sensory integration therapies.

Since all participants in the present study were young adults, it could from a developmental perspective still be possible that sensory difficulties rather than attention problems are primary to MSI abnormalities during childhood. Sensory difficulties might be present early on during development, but fade away with age, due to several compensation mechanisms. The present data cannot answer these questions and future studies should look at development of MSI in children with ASD. Further, the selective attention conditions chosen were directed to the visual modality only. Some argue that there is a bias toward this sensory modality in ASD [30]. In typically developing individuals it has been shown that sensory dominance can influence MSI effects [31]. Therefore, the presumed bias towards the visual modality might have interfered with the results. However, in our study we did not find group differences in the selective attention conditions. The fact that no group differences were found on the unisensory responses as well strengthens our claim that disruption of MSI in ASD is not related to differences in unisensory processing, but to mechanisms associated with cross-sensory divided attention.

### Conclusions

This is the first study to show the influence of attention on multisensory processing in individuals with ASD. The data clearly show that the multisensory processing of emotional signals in ASD is intact under appropriate circumstances. Atypical multisensory processing in ASD was shown to be secondary to attentional manipulation. The default pattern of information processing in individuals with ASD may lead to disruptive multisensory processing under naturalistic situations, and in this sense account for several features of the disorder. This might be relevant to treatment strategies, with training of multisensory attentional control possibly being more beneficial than conventional sensory integration therapy.
